# Genetic Modification Strategies to Enhance CAR T Cell Persistence for Patients With Solid Tumors

**DOI:** 10.3389/fimmu.2019.00218

**Published:** 2019-02-15

**Authors:** Christopher DeRenzo, Stephen Gottschalk

**Affiliations:** Department of Bone Marrow Transplantation and Cellular Therapy, St. Jude Children's Research Hospital, Memphis, TN, United States

**Keywords:** CAR T cell, immunotherapy, persistence, genetic modification, solid tumor

## Abstract

Immunotherapy with chimeric antigen receptor (CAR) T cells offers a promising method to improve cure rates and decrease morbidities for patients with cancer. In this regard, CD19-specific CAR T cell therapies have achieved dramatic objective responses for a high percent of patients with CD19-positive leukemia or lymphoma. Most patients with solid tumors however, have experienced transient or no benefit from CAR T cell therapies. Novel strategies are therefore needed to improve CAR T cell function for patients with solid tumors. One obstacle for the field is limited CAR T cell persistence after infusion into patients. In this review we highlight genetic engineering strategies to improve CAR T cell persistence for enhancing antitumor activity for patients with solid tumors.

## Introduction

**Figure 1:**
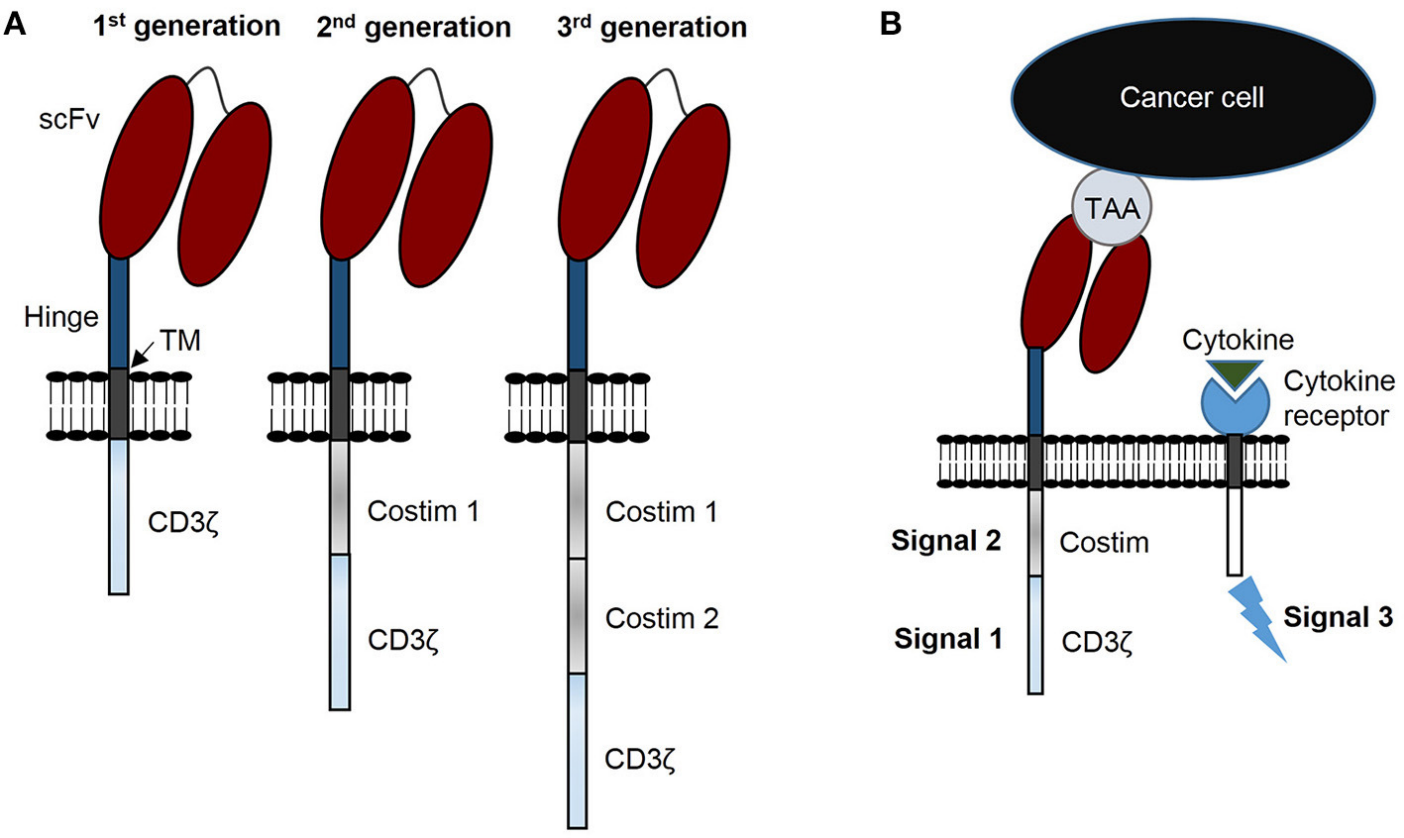
CAR terminology: components, generations, and signals. **(A)** CARs consist of 3 components: a single chain variable fragment (scFv) that recognizes tumor associated antigen (TAA), costimulatory domain(s), and a CD3ζ domain. CARs are designated 1st generation with no costimulatory domain, 2nd generation with 1 costimulatory domain, and 3rd generation with 2 costimulatory domains. **(B)** CAR T cells require 3 signals for optimal function: signal 1 is CD3ζ induced activation, signal 2 costimulation, and signal 3 functional augmentation via stimulatory cytokine(s). TM, transmembrane domain.

Patients with relapsed or refractory solid tumors have poor outcomes and novel treatments are needed to improve survival and decrease morbidities resulting from conventional treatments. Immunotherapy with chimeric antigen receptor (CAR) T cells is a promising strategy to improve these outcomes. Generally, CARs consist of three components: (i) a genetically engineered receptor capable of recognizing a tumor associated antigen (TAA) in an HLA-independent manner, (ii) an activation domain derived from the CD3ζ chain of the T cell receptor complex, and (iii) costimulatory domain(s) to potentiate activation initiated via CAR-CD3ζ signaling (Figure 1A). CARs are categorized as 1st generation if the construct has no costimulatory domain, 2nd generation when incorporating 1 costimulatory domain, and 3rd generation when incorporating two costimulatory domains (Figure 1A) (1). CAR T cells require three signals for optimal effector function: (i) activating signal induced by CAR recognition of tumor antigen and subsequent signal transduction through CD3ζ, (ii) costimulation provided via one or more domains engineered into the CAR construct, and (iii) stimulatory cytokines for continued growth and effector function (Figure 1B).

While CAR T cell therapy offers promise to improve outcomes for patients with difficult to treat cancers, strategies must be improved to benefit large numbers of patients suffering from relapsed and refractory solid tumors. Several obstacles need to be addressed to realize this goal, including selection of optimal TAAs (2–4), T cell homing to sites of malignant disease (5, 6), T cell penetration into solid masses (7, 8), and overcoming the immunosuppressive tumor microenvironment (9–11). Enhancing persistence of adoptively transferred T cells is another vital challenge for successful treatment of cancer (12–14). Multiple strategies exist to enhance CAR T cell persistence against solid tumors including pre-treatment with cytoreductive chemotherapy (15), optimized T cell culture conditions (16), T cell selection procedures (17, 18), and combinatorial treatment approaches (19–24). This review is focused on novel genetic engineering strategies to enhance CAR T cell persistence and antitumor activity against solid tumors. While promising preclinical data is available, only few of these approaches have been evaluated in early phase clinical studies.

## Novel Costimulation Strategies to Improve Persistence and Anti-Solid Tumor Activity

Second generation CAR constructs with CD28 or 41BB costimulatory endodomains are most frequently utilized to generate CAR T cells. Comparison of CD28 vs. 41BB to enhance the function of CAR T cells has been reviewed previously (25). Investigators are now actively exploring CARs encoding alternative costimulatory endodomains or strategies to provide costimulation with a 2nd molecule expressed in CAR T cells.

**Figure 2:**
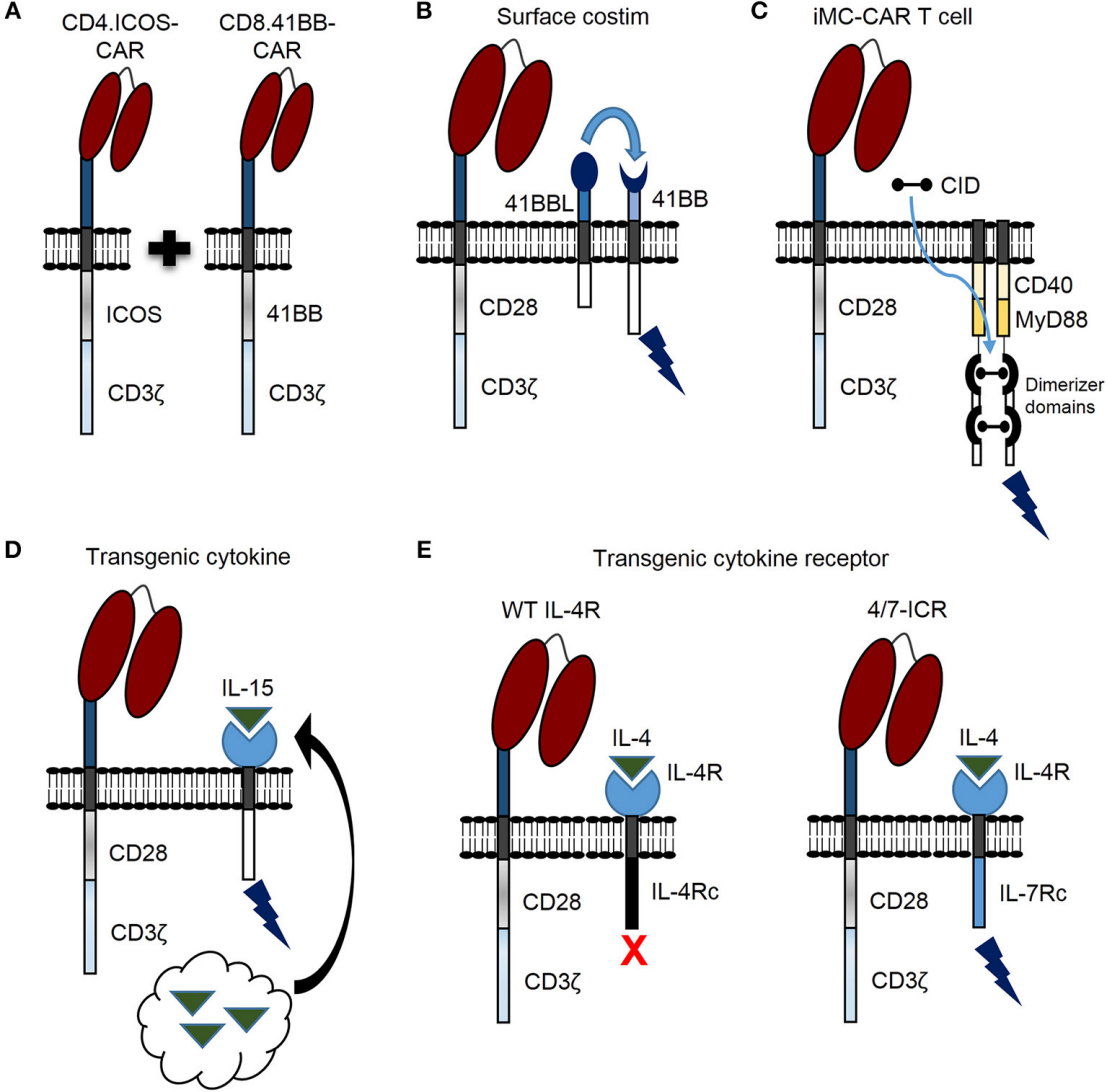
Novel genetic modifications to enhance CAR T cell persistence against solid tumors. **(A)** CAR T cell persistence against solid tumors can be enhanced by transducing CD4 and CD8 T cells with different CAR costimulatory domains. **(B)** CAR T cell activation leads to 41BB receptor (41BB) expression. CAR T cell persistence can be enhanced by constitutive expression of 41BB ligand (41BBL), which interacts with the 41BB receptor in an autocrine or paracrine manner to provide additional costimulatory signal (surface costim). **(C)** Triggering CAR T cell costimulation with a chemical inducer of dimerization (CID) drug is another promising approach. A molecule, which consists of two dimerizer domains and costimulatory domains derived from MyD88 and CD40 (iMC) can be activated by CID and enhance CAR T cell persistence. **(D)** Constitutively expressed cytokine (i.e., IL-15) can be engineered by tethering cytokine to the cell membrane (not shown) or secreted by CAR T cells (black arrow) to enhance persistence. **(E)** The inverted IL-4/IL-7 chimeric cytokine receptor (4/7-ICR) contains an IL-4 receptor extracellular domain (IL-4R) fused to an activating IL-7 cytoplasmic domain (IL-7Rc). While IL-4 binding to wild type (WT) IL-4 receptor leads to CAR T cell inhibition, IL-4 binding to 4/7-ICR enhances persistence and effector function.

Recent findings demonstrate that CAR T cell persistence can be enhanced against solid tumors by transducing CD4 and CD8 T cells with CARs encoding different costimulatory domains (Figure 2A) (26). This was demonstrated by separately transducing CD4 and CD8 T cells with mesothelin-specific CARs containing either a CD28, 41BB, or ICOS costimulatory domain. After separate transductions, CD4- and CD8-CAR T cells were mixed and infused to determine the optimal combination for enhanced persistence against lung cancer *in vivo*. Strikingly, the best combination for enhanced persistence of CD8-CAR T cells resulted from mixing CD4.ICOS- and CD8.41BB-CAR T cells before injection. CD4.ICOS-CAR T cells persisted *in vivo* regardless of the costimulatory domain expressed by CD8-CAR T cells. On the other hand, CD4-CAR T cells expressing either a CD28 or 41BB costimulatory domain had minimal persistence under any condition, clearly demonstrating that the costimulatory domain of CD4-CAR T cells affects persistence of both CD4- and CD8-CAR T cells in this model. Based on these data, the authors generated a 3rd generation ICOS.41BB-CAR, which also led to enhanced persistence of both CD4- and CD8-CAR T cells *in vivo*, and greater antitumor activity compared to 2nd generation CAR T cells. Given that CD4 T cells provide signals to enhance persistence and effector function of cytotoxic CD8 T cells, this concept is highly relevant for development of future CAR T cell trials for patients with solid tumors. Furthermore, evidence demonstrates that, at least in some models, CD4-CAR T cells targeting solid tumors persist longer *in viv*o and result in superior antitumor activity compared to CD8-CAR T cells (27). Given that optimal costimulatory domains may be different for CD4- and CD8-CAR T cells, CD4-CAR T cells can directly kill cancer cells, and single vectors can be used to make 3rd generation CARs for enhancing persistence of both CD4- and CD8-CAR T cells, future studies focused on optimizing costimulation for both CD4- and CD8-CAR T cells should benefit patients with solid tumors.

Providing costimulation through a 2nd molecule is another promising strategy to enhance persistence, antitumor activity, and safety of CAR T cells for patients with solid tumors. For example, activated T cells express the 41BB receptor, and investigators have shown that CD28-CAR T cells expressing 41BB ligand on the cell surface (Figure 2B) endows T cells with superior effector function in comparison to T cells expressing a traditional 3rd generation CD28.41BB-CAR (28). Expressing other tumor necrosis factor superfamily ligands on the T cell surface, such as CD40 ligand, has also resulted in improved CAR T cell function (29).

Triggering CAR T cell costimulation with a drug is another promising approach. For example, investigators designed a molecule, which consists of two dimerizer domains and costimulatory domains derived from MyD88 and CD40 (iMC) (Figure 2C) and can be activated by a chemical inducer of dimerization (CID) (30). While initially, this molecule was used to activate dendritic cells, two subsequent studies, as delineated in the next section, have highlighted the benefit of iMC in CAR T cells (31, 32). Of note, costimulation is only activated in the presence of CID, providing a safety mechanism to limit T cell activation.

Using 1st generation prostate-specific antigen (PSCA)-CAR T cells containing an iMC domain (PSCA-iMC-CAR) and PSCA-positive pancreatic cancer cells *in vitro*, Foster and colleagues demonstrated that CID induced costimulation resulted in greater CAR T cell cytokine secretion, proliferation, and antitumor activity compared to the same cells without CID. *In vivo*, PSCA.iMC-CAR T cells were used to treat mice harboring pancreatic cancers plus/minus weekly CID. Indeed, CID administration enhanced PSCA.iMC-CAR antitumor activity, and 21 days post T cell injection CAR T cells were detected in the blood and tumors from mice treated with CID, but not in those treated without CID.

Separately, Mata and colleagues generated HER2-specific CAR T cells incorporating an inducible costimulatory domain (HER2.iMC-CAR) containing both MyD88 and CD40 signaling regions, (32) and demonstrated that HER2.iMC-CAR T cells have enhanced effector function compared to 2nd generation HER2.CD28-CAR T cells utilized in clinical trials (33). Importantly, in repeat stimulation assays, which model CAR T cell persistence against solid tumors *in vitro*, HER2.iMC-CAR T cells expanded 100–1,000 fold greater than 2nd generation HER2-CAR T cells when targeting multiple different HER2-positive solid tumor types. *In vivo*, HER2.iMC-CAR T cells had significantly greater antitumor activity that led to an enhanced survival benefit compared to 2nd generation HER2-CAR T cells, and controlled solid tumors at a very low T cell dose. Furthermore, repeated induction of costimulation via multiple doses of CID led to superior antitumor activity and a survival advantage for mice bearing HER2-positive solid tumors.

In summary, these results demonstrate that non-traditional costimulatory domains can enhance CAR T cell persistence and antitumor activity against solid tumors, and an inert drug can be injected to adjust CAR T cell activation over time.

## Transgenic Cytokines Enhance CAR T Cell Effector Function Against Solid Tumors

In addition to modifying costimulatory domains, genetic modification can be used to endow CAR T cells with the ability to express stimulatory cytokines. Interleukin (IL)-15, IL-12, and IL-18 are three examples under active exploration. Constitutively expressed IL-15 can be engineered by tethering IL-15 to the cell membrane (34) or secreted by modified T cells (Figure 2D) (35). In this regard, IL-13Rα2-CAR T cells modified to secrete IL-15 (IL-13Rα2.IL-15-CAR) demonstrated superior proliferative capacity and antitumor activity *in vitro* and *in vivo* against high grade glioma compared to IL-13Rα2-CAR alone (35). Both IL-13Rα2- and IL-13Rα2.IL-15-CAR T cells had comparable *in vivo* antitumor activity up to 4 weeks; however, after 4 weeks IL-15 expressing CAR T cells had greater activity indicating that IL-15 improved T cell persistence over a prolonged period of time. Indeed, IL-15 expressing CAR T cells were detected *in vivo* for a significantly longer period of time compared to CAR alone. Intriguingly, in mice treated with IL13-Rα2.IL-15-CAR T cells, tumors recurred at late time points and the majority of relapsed tumors no longer expressed IL-13Rα2, implicating antigen loss as a tumor escape mechanism in this model. This predicts that despite the benefits of improving CAR T cell persistence against solid tumors, antigen loss variants can occur, and strategies to target solid tumors in future clinical trials may require targeting multiple tumor antigens (36, 37). Clinically, transgenic IL-15 expression is actively being explored to improve expansion, persistence and antitumor activity of GD2-CAR invariant natural killer cells for the treatment of patients with neuroblastoma (NCT03294954). Results from this trial should provide insight regarding the impact of constitutively secreted IL-15 to enhance persistence and function of adoptively transferred CAR modified cells, and determine safety in the clinical setting.

IL-12 is another promising cytokine under active exploration to enhance CAR T cell persistence and effector function in both preclinical models (38–40) and a phase I clinical trial for patients with solid tumors (NCT02498912). To enhance CAR T cell activity against ovarian cancer, 2nd generation MUC16^ecto^-specific CAR T cells were modified to secrete IL-12 (MUC16^ecto^.IL-12-CAR) (40). *In vivo* MUC16^ecto^.IL12-CAR T cells demonstrated superior antitumor activity and were detected in the peripheral blood of treated animals, while the same CAR T cells without IL-12 were not detected at any time point, indicating that constitutive IL-12 secretion increased CAR T cell persistence against ovarian cancer. A clinical trial is underway investigating MUC16^ecto^.IL-12-CAR T cells for patients with MUC16^ecto^-positive tumors (NCT02498912), and results should shed light on the possibility of translating this technique to treat a broad range of patients afflicted with solid tumors.

CAR T cells genetically modified to secrete IL-18 exhibit superior antitumor activity against solid tumors compared to 2nd generation CAR T cells in pre-clinical models. Chmielewski and Abken compared 2nd generation CEA-CAR T cells containing a CD28 costimulatory domain to CEA-CAR T cells modified to secrete IL-18 (CEA.IL-18-CAR) under control of a nuclear factor of activated T cells (NFAT)-IL-2 minimal promoter (41). Placing cytokine secretion under control of the NFAT-IL-2 promoter creates an inducible system, whereas cytokine is only secreted upon T cell recognition of its target antigen, theoretically limiting cytokine secretion to the tumor environment. In an immune-competent model of bulky CEA-positive pancreatic cancer, a single injection of CEA.IL-18-CAR T cells led to prolonged survival compared to mice treated with 2nd generation CEA-CAR. Prolonged survival and enhanced antitumor activity were attributed to a pro-inflammatory environment induced by CAR mediated IL-18 secretion. Compared to tumors treated with 2nd generation CEA-CAR, tumors obtained after CEA.IL-18-CAR treatment demonstrated an increased quantity of pro-inflammatory natural killer cells and M1 macrophages, and a decreased quantity of anti-inflammatory M2 macrophages, regulatory T cells, and CD103-positive dendritic cells. Other groups have shown enhanced antitumor activity by genetically modifying T cells to secrete IL-18 (42, 43), and this strategy merits further exploration to enhance CAR T cell activity against solid tumors.

Stimulatory cytokine pathways can also be constitutively activated without the need for cytokine induced stimulation, thus providing T cell survival signals when no cytokine is in the milieu. To enhance expansion, persistence and antitumor activity of 2nd generation GD2-CAR T cells against neuroblastoma, investigators modified CAR T cells with a constitutively active IL-7 cytokine receptor (C7R) that lacks the IL-7 receptor extracellular domain (44). C7R modified CAR T cells were able to proliferate and kill neuroblastoma cells in serial killing assays to a greater degree than GD2-CAR T cells alone. Impressively, at a low T cell dose GD2.C7R-CAR T cells had substantial antitumor activity *in vivo* against metastatic neuroblastoma. Comparatively, GD2-CAR T cells had limited antitumor activity at the same low T cell dose. Improved antitumor activity resulted in enhanced survival that was secondary to increased expansion and persistence of C7R expressing CAR T cells. Mechanistically, GD2-CAR T cells expressing C7R had greater cell division and reduced apoptosis compared to GD2-CAR alone, effects attributed, in part, to increased BCL2 expression.

While CAR T cell transgenic cytokine production and signaling offer a promising strategy to improve persistence and function of CAR T cells for patients with solid tumors, safety concerns exist. T cell autonomous growth (45) and cytokine induced toxicity can occur (46), and these concerns need to be considered for treating patients. Notably, for both IL-13Rα2.IL15-CAR and MUC16^ecto^.IL-12-CAR T cells, IL-15 or IL-12 production was low at baseline and increased multiple fold in an antigen specific manner, indicating that elevated cytokine levels should be limited to the local tumor environment. Additionally, both aforementioned CARs were modified with a “safety switch” that proved capable of efficiently eliminating gene modified cells. IL-13Rα2.IL-15-CAR T cells were modified with an inducible caspase 9 (iC9) safety switch that initiates apoptosis in the presence of CID, and these T cells were efficiently eliminated by CID induced iC9 activation. Importantly, the iC9 safety switch was demonstrated to be effective in the clinical setting (47) making it a viable safety mechanism for future trials. MUC16^ecto^.IL-12-CAR T cells were modified with a truncated epidermal growth factor receptor that can be targeted by cetuximab, a clinically available monoclonal antibody.

As discussed previously, IL-18 secretion in CEA-CAR T cells was under control of an NFAT-IL-2 minimal promoter, which enables T cells to secrete IL-18 only in the presence of target antigen. For CEA.IL-18-CAR T cells, IL-18 was secreted at high levels in the presence of CEA-positive pancreatic cancer cells, but only minimal IL-18 was detected when T cells were exposed to CEA-negative pancreatic cancer cells. Although this strategy demonstrated benefit in this and other pre-clinical studies (48), initial experience in humans was less promising. In one clinical study, the adoptive transfer of *ex vivo* expanded tumor infiltrating lymphocytes transduced to secrete IL-12 under the control of an NFAT-IL-2 minimal promoter induced significant toxicities attributed to high serum IL-12 levels in patients with melanoma (49).

In regard to safety for C7R modified CAR T cells, these demonstrated no cell autonomous growth, and included an iC9 safety switch that led to efficient GD2.C7R-CAR T cell elimination when treated with CID. Alternate gene modification strategies can also be utilized to enhance the safety of CAR T cells by enabling full CAR T cell activation only in the presence of 2 TAAs. Examples include chimeric costimulatory receptors (50, 51) and synthetic Notch receptors (52). Another novel method is to simultaneously induce CAR T cell STAT5 signaling, analogous to signal provided by IL-2, IL-7, or IL-15, and STAT3 signaling, analogous to signal provided by IL-21, in an antigen dependent manner. In this regard, Kagoya and colleagues developed a “new generation” CD19-CAR containing a CD28 costimulatory domain, a truncated IL-2Rβ domain to activate STAT5, and a C-terminus YXXQ motif to activate STAT3 (53). Importantly, STAT5 and STAT3 activation occurred in an antigen dependent manner and the new generation CAR enhanced proliferation of both CD4- and CD8-CAR T cells *in vitro* and *in vivo* compared to 2nd generation CAR T cells with either CD28 or 41BB costimulatory domains. In an *in vivo* solid tumor model using A375 melanoma cells modified to express CD19, new generation CAR T cells persisted to a greater degree in tumors and peripheral blood of treated animals, and had greater antitumor activity compared to 2nd generation CAR T cells.

Together, these findings demonstrate that while safety concerns exist, strategies can be implemented to eliminate genetically modified cells in the setting of unacceptable toxicity, limit full CAR T cell activation to sites of tumor expressing two or more TAAs, or deliver cytokine signals to CAR T cells via activation of STAT5 and STAT3 in an antigen dependent manner.

In summary, studies demonstrate that CAR T cells can be safely engineered to express transgenic cytokines or constitutively active cytokine receptors, which impart CAR T cells with enhanced persistence and antitumor activity against solid tumors. Active and future clinical trials implementing these techniques will guide strategies to improve outcomes for patients with solid tumors.

## Transgenic Cytokine Receptors to Overcome Tumor Secreted Inhibitory Milieu

Overcoming effects of inhibitory cytokines is another strategy to enhance CAR T cell persistence and function for treating patients with solid tumors. Switch receptors are one way to transform inhibitory cytokine signals into a stimulus, and thus increase T cell persistence and function (Figure 2E) (54, 55). Effectiveness of this strategy was demonstrated against pancreatic cancer using a PSCA-specific CAR engineered with an inverted IL-4/IL-7 chimeric cytokine receptor (4/7-ICR) (56), which contains an IL-4 extracellular domain fused to an activating IL-7 intracellular domain. PSCA-CAR T cells initially killed PSCA-positive pancreatic cancer cells, but only PSCA.4/7-ICR-CAR T cells killed and expanded in the presence of both pancreatic cancer cells and the inhibitory cytokine IL-4. This held true *in vivo* where PSCA.4/7-ICR-CAR T cells exhibited enhanced antitumor activity and greater expansion compared to CAR T cells without 4/7-ICR. Importantly, once PSCA-positive solid tumor cells were eliminated, PSCA.4/7-ICR-CAR T cells no longer expanded, indicating no cell autonomous growth and a positive safety profile. This strategy was also utilized to target breast cancer using 2nd generation MUC1-CAR T cells (57). MUC1.4/7-ICR-CAR T cells demonstrated enhanced expansion and antitumor activity *in vitro* and *in vivo* compared to MUC1-CAR alone, demonstrating that the 4/7-ICR enhances CAR T cell persistence and function against multiple solid tumor types in the presence of the inhibitory cytokine IL-4.

Transgenic cytokine receptors can also be combined to impart CAR T cells with the ability to transform multiple inhibitory signals into different types of T cell stimuli. Transgenic cytokine receptors were previously developed to overcome the inhibitory effect of transforming growth factor β (TGFβ), a potent immunosuppressive cytokine secreted by multiple solid tumor types. Dominant negative TGFβ receptors (DNR) enable T cells to avoid effects exerted by TGFβ (58, 59), and DNR transduced tumor-specific T cells were recently shown to safely persist in patients years after T cell infusion (60). TGFβ receptor II extracellular domains can also be fused with stimulatory intracellular domains such as the 41BB endodomain (TGFβ/41BB). In a recent publication 1st generation PSCA-CAR T cells were modified to express both TGFβ/41BB and 4/7-ICR, and dubbed SmarT cells (61). SmarT cells demonstrated the ability to recognize PSCA antigen on pancreatic cancer cells through the CAR, induce costimulation via TGFβ induced activation of 41BB, and initiate cytokine signaling through 4/7-ICR in the presence of IL-4. Triple genetic modification enabled SmarT cells to specifically recognize PSCA-positive pancreatic cancer cells, expand, persist, and kill tumor cells in an immunosuppressive TGFβ and IL-4 rich environment. Importantly continued SmarT cell expansion and antitumor activity were dependent upon both PSCA antigen positivity and cytokine induced stimulation through the transgenic receptors. *In vivo*, SmarT cells expanded, persisted, and eliminated pancreatic tumors expressing PSCA, TGFβ, and IL-4, with minimal expansion/persistence at tumor sites expressing PSCA only. Once PSCA-positive, TGFβ, and IL-4 secreting solid tumors were eliminated, SmarT cells contracted, further demonstrating safety of this approach.

In conjunction with enhancing CAR T cell persistence and effector function, transgenic cytokine receptors can be used for selectively enhancing growth of transduced CAR T cells during the T cell manufacturing process. In this regard, a 4αβ chimeric cytokine receptor was developed, consisting of an extracellular IL-4Rα domain paired with an endodomain derived from the shared IL-2/IL-15 β chain (62). This strategy was demonstrated to be effective for generating CAR T cells to target multiple solid tumor types in pre-clinical studies (20, 63), and is under active investigation in a clinical trial for patients with head and neck squamous cell carcinoma (64) (NCT01818323).

In summary, transgenic cytokine receptors can be utilized to avoid immunosuppressive effects of inhibitory cytokines, transform inhibitory signals into activating signals, and to selectively grow transduced solid tumor specific CAR T cells.

## Challenges Ahead

We have described novel genetic modification strategies to enhance the expansion and persistence of CAR T cells for patients with solid tumors. The next frontier in this regard is to overcome limitations imposed by current gene modification techniques, and endow CAR T cells with even greater functional capacity. CAR T cells face many obstacles including homing to and penetrating into solid masses, overcoming tumor antigen escape, surviving within the hostile tumor environment of low pH, high lactate and adenosine, hypoxia, and numerous other immunosuppressive factors including inhibitory stromal cells. Realistically, current engineering strategies allow up to 3 genetic modifications to enhance CAR T cell function. While individual genetic modifications demonstrate efficacy to combat many of the listed challenges, the field is tasked with developing new strategies to combine a greater number of mechanisms for enabling individual CAR T cell products to overcome the potent solid tumor inhibitory environment. One intriguing strategy is to physically load CAR T cells with nanogel “backpacks” capable of delivering relatively large quantities of protein upon CAR recognition of target antigen (65). In such a paradigm, CAR T cells could be physically loaded with proliferative cytokines, leaving room to utilize genetic modifications to overcome other obstacles, such as targeting multiple solid tumor antigens. Another promising strategy is to utilize targeted gene editing and insert CAR constructs into T cell inhibitory loci (66, 67). For instance, CAR DNA could be inserted into the adenosine 2a receptor locus (68), decreasing CAR T cell immunosuppression in an adenosine rich milieu. As these, and other novel strategies are implemented, we envision CAR T cells capable of safely persisting long term and overcoming a greater number of tumor immune evasion strategies.

## Conclusions

Several strategies have been developed to enhance CAR T cell expansion, persistence and antitumor activity by introducing novel costimulatory domains, cytokine genes, and constitutively active or inverted cytokine receptors into T cells. While there are safety concerns regarding autonomous cell growth and cytokine induced toxicity using these approaches, encouraging efficacy and safety data from preclinical studies supports continued preclinical testing and evaluation in humans. Thus, we remain hopeful that optimized CAR T cells will eventually improve outcomes and decrease toxicities for patients suffering from solid tumors.

## Author Contributions

All authors listed have made a substantial, direct and intellectual contribution to the work, and approved it for publication.

### Conflict of Interest Statement

SG has patent applications in the field of cell and gene therapy for cancer, and receives research support from TESSA Therapeutics. The remaining author declares that the research was conducted in the absence of any commercial or financial relationships that could be construed as a potential conflict of interest.
